# Avian H6 Influenza Viruses in Vietnamese Live Bird Markets during 2018–2021

**DOI:** 10.3390/v16030367

**Published:** 2024-02-27

**Authors:** Lizheng Guan, Lavanya Babujee, Robert Presler, David Pattinson, Hang Le Khanh Nguyen, Vu Mai Phuong Hoang, Mai Quynh Le, Harm van Bakel, Yoshihiro Kawaoka, Gabriele Neumann

**Affiliations:** 1 Influenza Research Institute, Department of Pathobiological Sciences, School of Veterinary Medicine, University of Wisconsin—Madison, Madison, WI 53711, USA; lizheng.guan@wisc.edu (L.G.); lavanya.babujee@wisc.edu (L.B.); david.pattinson@wisc.edu (D.P.); 2 National Institute of Hygiene and Epidemiology, Hanoi 100000, Vietnam; nlkh@nihe.org.vn (H.L.K.N.); hvmp@nihe.org.vn (V.M.P.H.); ltqm@nihe.org.vn (M.Q.L.); 3 Department of Genetics and Genomic Services, Icahn School of Medicine at Mount Sinai, New York, NY 10029, USA; harm.vanbakel@mssm.edu; 4 Division of Virology, Department of Microbiology and Immunology, and International Research Center for Infectious Diseases, The Institute of Medical Science, University of Tokyo, Tokyo 108-8639, Japan; 5 Research Center for Global Viral Diseases, National Center for Global Health and Medicine, Tokyo 162-8655, Japan; 6 Infection and Advanced Research (UTOPIA) Center, The University of Tokyo Pandemic Preparedness, Tokyo 108-8639, Japan

**Keywords:** surveillance, H6, influenza virus, reassortment, Vietnam

## Abstract

Avian influenza viruses of the H6 subtype are prevalent in wild ducks and likely play an important role in the ecology of influenza viruses through reassortment with other avian influenza viruses. Yet, only 152 Vietnamese H6 virus sequences were available in GISAID (Global Initiative on Sharing All Influenza Data) prior to this study with the most recent sequences being from 2018. Through surveillance in Vietnamese live bird markets from 2018 to 2021, we identified 287 samples containing one or several H6 viruses and other influenza A virus subtypes, demonstrating a high rate of co-infections among birds in Vietnamese live bird markets. For the 132 H6 samples with unique influenza virus sequences, we conducted phylogenetic and genetic analyses. Most of the H6 viruses were similar to each other and closely related to other H6 viruses; however, signs of reassortment with other avian influenza viruses were evident. At the genetic level, the Vietnamese H6 viruses characterized in our study encode a single basic amino acid at the HA cleavage site, consistent with low pathogenicity in poultry. The Vietnamese H6 viruses analyzed here possess an amino acid motif in HA that confers binding to both avian- and human-type receptors on host cells, consistent with their ability to infect mammals. The frequent detection of H6 viruses in Vietnamese live bird markets, the high rate of co-infections of birds with different influenza viruses, and the dual receptor-binding specificity of these viruses warrant their close monitoring for potential infection and spread among mammals.

## 1. Introduction

The first avian influenza virus of the H6 subtype was detected in 1963 (A/turkey/Canada/1963, H6N8), but it was not until 1976 that viruses of the H6 subtype were reported in Asia. Viruses of the H6 subtype are now enzootic in wild birds in Asia and represent the most detected subtype of low pathogenic avian influenza viruses in ducks (www.gisaid.org; accessed on 27 October 2023). Phylogenetic analyses, including those for H6 viruses (reviewed in [1]), have demonstrated frequent reassortment among avian influenza viruses. One important example is the highly pathogenic H5 A/goose/Guangdong/1/1996 virus lineage whose internal genes are closely related to those of the H6 virus A/teal/Hong Kong/W312/97 [2]. H6 virus genes also share similarity with the genes of some highly pathogenic H7N9 influenza viruses [3]. In addition, there have been reports of H6 virus infection of a human in 2013 [4] and of pigs [5,6] and a dog [7], demonstrating their ability to infect mammals.

Phylogenetic analysis of the hemagglutinin (HA) gene indicated that a single lineage of H6 viruses was circulating in live bird markets in Hong Kong until the early 2000’s, represented by the A/teal/Hong Kong/W312/97 (W312) virus [8]. A detailed phylogenetic analysis of the HA genes of H6 viruses isolated from domestic ducks in Southern China from 2000 to 2005 revealed three additional lineages: Group I (represented by A/duck/Shantou/339/2000), Group II (represented by A/wild duck/Shantou/2853/2003), and Group III (represented by A/duck/Hunan/573/2002) [9]. Group I comprised viruses from Guangdong and Hong Kong, mostly possessing a neuraminidase (NA) gene of the N2 subtype [9]. Group II included H6N2 and H6N6 viruses from the Guangdong and Fujian provinces [9], whereas viruses in Group III came from Jiangxi, Hunan, and Guangxi and possessed Nas of different subtypes [9]. In later years, most of the analyzed Chinese H6 viruses belonged to Groups I and II, with an increasing percentage of viruses possessing an NA gene of the N6 subtype [10,11,12,13]. Moreover, analysis of recent Asian H6 viruses revealed frequent reassortment with other influenza A viruses, resulting in multiple genotypes that have been co-circulating [14,15,16,17].

H6 viruses replicate efficiently in mice without prior adaptation [15,18,19,20,21,22]. Typically, H6 virus infections do not cause severe disease in mice [15,19,20,21,23], but in rare cases, systemic spread [14] and death [17] have been reported. In guinea pigs, inefficient transmission to animals housed in the same cage was observed [21].

Typically, avian influenza viruses (including H6 viruses) preferentially bind to α2,3-linked sialic acids (i.e., avian-type receptors), which are expressed on epithelial cells in the intestinal tract of birds. However, many H6 viruses also bind to α2,6-linked sialic acids (i.e., human-type receptors) [15,16,21,24,25,26], which are predominant on epithelial cells in the respiratory organs of humans. The receptor-binding specificity of influenza viruses is mainly determined by amino acids at and around the receptor-binding site. For H6 HAs, structural and experimental analyses have identified several amino acid positions that affect receptor-binding specificity, including HA amino acid positions 137, 190, 222, 225, 226, and 228 (for consistency with the published literature, the H6 HA numbering from Ni et al. [27] will be used throughout) [1,24,25,27,28]. Specifically, the HA-G225D [28], HA-Q226L [25], and HA-G228S [25] substitutions confer human-type receptor-binding properties to an H6 virus, and an H6 virus encoding HA-226L was found to have acquired the ability to transmit through respiratory droplets in guinea pigs [25].

Prior to our study, only 152 Vietnamese H6 virus sequences were available in GISAID (www.gisaid.org, accessed on 9 September 2023); of those 152, the most recent were from 2018 and belong to Group II [29,30]. During our 2018 to 2021 surveillance in Vietnamese live bird markets, we identified 287 samples that contained at least one H6 virus. More than half of them contained sequences from at least one other influenza virus of the same or different HA subtype, demonstrating a high rate of co-infection of birds with different influenza viruses. Phylogenetic and deep-sequencing analyses placed the isolated viruses into Group II (represented by A/wild duck/Shantou/2853/2003) [9] and identified amino acid substitutions that might affect their viral properties. Collectively, our data increase our understanding of the H6 viruses circulating in Vietnam in recent years.

## 2. Materials and Methods

### 2.1. Virus Isolation and Identification

The National Institute of Health and Epidemiology of Vietnam conducts routine surveillance activities in live bird markets in two provinces of Northern Vietnam, Ha Noi and Quang Ninh. During these activities, 3060 oropharyngeal and cloacal swab samples (including the 2430 described in our previous publication [31]) were collected from apparently healthy birds between 2018 and 2021. All samples were stored in transport medium (DMEM containing 0.15% BSA, 100 IU/mL of penicillin-streptomycin, 0.5 μg/mL of amphotericin B, 100 μg/mL of gentamicin, 20 μg/mL of ciprofloxacin, and 0.02 M of HEPES) until further processing.

First, pooled samples were inoculated into 9–11-day-old specific pathogen-free (SPF) eggs. Specifically, we combined up to 10 samples that were collected in the same location from the same species on the same day. Inoculated eggs were incubated at 35 °C for 24–48 h, and the allantoic fluid was then tested for hemagglutination activity. From pools with hemagglutination activity, all individual samples were then inoculated into two SPF eggs each, followed by testing for hemagglutination. All hemagglutination-positive samples were then characterized by deep-sequencing analysis.

### 2.2. Viral Genomic Sequence Analysis

We used the MagMAXTM-96 Viral RNA Isolation Kit (ThermoFisher, Waltham, MA, USA) to extract RNAs, which were then amplified by RT-PCR with the following oligonucleotides: F1 (5′-GTTACGCGCCAGCAAAAGCAGG); F2 (5′-GTTACGCGCCAGCGAAAGCAGG); and R1 (5′-GTTACGCGCCAGTAGAAACAAGG). The purification of the amplified double-stranded cDNAs was carried out with 0.45× volume of AMPure XP beads (Beckman Coulter, Brea, CA, USA), followed by acoustic shearing (0.5–1 μg) on a Bioruptor Pico sonicator (Diagenode, Denville, NJ, USA) with an average fragment size of 150 bp. Libraries for next-generation sequencing were generated by using the NEBNext DNA library prep modules for Illumina (New England Biolabs, Ipswich, MA, USA).

The Illumina MiSeq demultiplexed reads were processed and assembled by using the Iterative Refinement Meta-assembler (IRMA v. 1.0.2) [32]. The sequences of all influenza viral genes were obtained from the primary assembly, except for the HA and NA sequences, which were obtained from the secondary assembly. Default IRMA FLU parameters were used, with the following exceptions: LABEL was used for read sorting, the residual assembly factor was set to 400 for the secondary assembly, and reference elongation was prevented. All sequences were then analyzed by using version v0.9.9 of a snakemake workflow available at https://github.com/IRI-UW-Bioinformatics/flu-ngs/releases/tag/v0.9.9. Database accession numbers are provided in Supplementary Table S1.

### 2.3. Phylogenetic Analysis

Influenza virus sequences from avian hosts collected in Asia between 1 January 1995 and 24 July 2023 were obtained from GISAID (www.gisaid.org; accessed on 9 September 2023) (Supplementary Table S2). For HA, the sequences from all H6 viruses were downloaded. For NA, all N6 sequences were downloaded. For the remaining viral genes, all influenza A virus sequences were used. The downloaded sequences were filtered using seqkit [33] to remove short (less than 80% full length) and redundant sequences. For HA and NA, all the sequences retained after filtering were used to construct phylogenetic trees. For each of the other viral RNA segments, 500 sequences were randomly selected from the filtered set using seqkit for inclusion in phylogenetic trees. Surveillance sequences were included after filtering out for redundancy. The sequences were aligned using MAFFT [34]. Maximum likelihood phylogenetic trees were constructed using RAxML-ng [35] with the GTR+I+G4 substitution model. Up to 1000 bootstrap replicates were conducted using MRE-based bootstopping [36]. Trees were visualized using ggtree [37]. A/turkey/Minnesota/957/80 (H6N6) was an outgroup used to root the trees. If surveillance isolates possessed the same nucleotide sequence in an influenza viral RNA segment, only one isolate is shown in the phylogenetic trees. Isolates with identical sequences are listed in Supplementary Table S3.

### 2.4. Biosafety Statement

Surveillance samples were isolated in an enhanced biosafety level 3 (BSL 3+) laboratory at the University of Wisconsin–Madison. The RNA extraction/inactivation protocol was approved by the University of Wisconsin–Madison’s Institutional Biosafety Committee (IBC) after conducting a risk assessment in the Office of Biosafety. All experiments were approved by the University of Wisconsin–Madison’s IBC. The NIAID grants for the studies conducted were reviewed by the University of Wisconsin–Madison Dual Use Research of Concern (DURC) Subcommittee in accordance with the United States Government September 2014 DURC Policy and determined to not meet the criteria of DURC. The University of Wisconsin–Madison Institutional Contact for Dual Use Research reviewed this manuscript and confirmed that the studies described herein do not meet the criteria of DURC.

## 3. Results

### 3.1. Viruses of the H6 Subtype Isolated from Vietnamese Live Bird Markets from 2018 to 2021

From 2018 through to 2021, we collected a total of 3060 oropharyngeal and cloacal swabs from apparently healthy ducks and chickens at live bird markets in two provinces of northern Vietnam ([31], this study). Pooled samples were amplified in embryonated chicken eggs and tested for hemagglutination activity. If a pooled sample showed hemagglutination activity, all samples used to generate the pooled sample were individually inoculated into embryonated chicken eggs and tested for hemagglutination. We then deep-sequenced the whole genomes of these viruses. In total, 287 samples contained an H6 virus, but more than half of them (i.e., 155 samples) contained a viral gene segment from more than one influenza virus. For the remaining 132 H6 virus samples (see Supplementary Table S1), we performed phylogenetic and sequence analyses.

### 3.2. Phylogenetic Analysis of H6 Viruses

**Figure 1:**
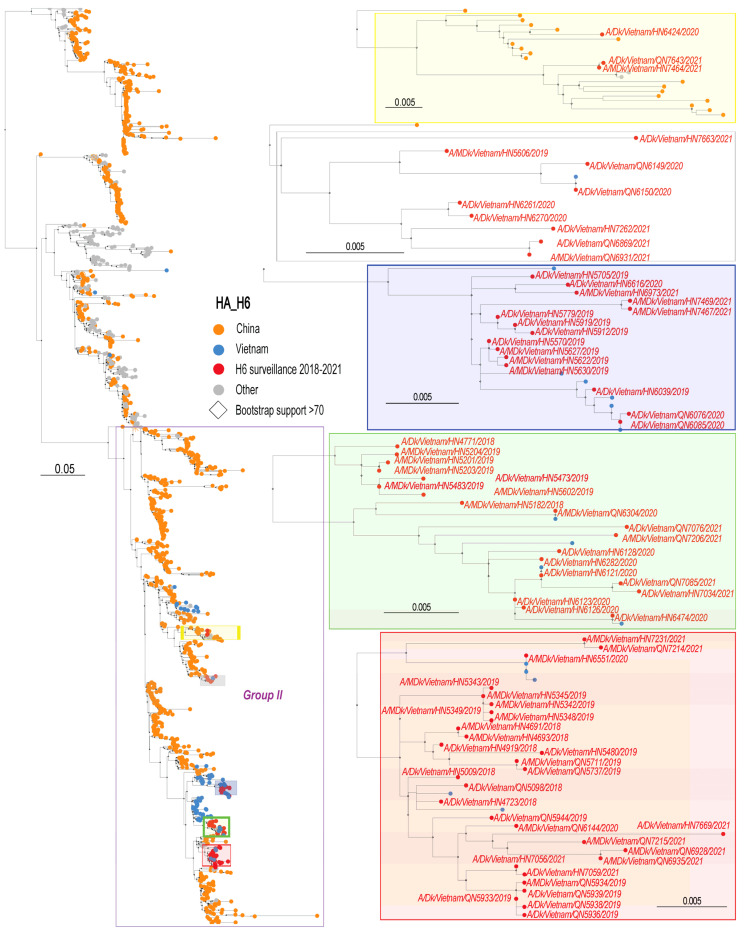
Phylogenetic analysis of the HA genes of H6 influenza viruses isolated in Vietnamese live bird markets from 2018 to 2021. Shown on the left is a schematic phylogenetic tree (Best ML tree, RAxML-NG 1000 bootstrap replicates) without virus names. Major phylogenetic H6 virus groups (i.e., W312 and Groups I, II, and III) are indicated. The sections of the phylogenetic tree containing H6 viruses from this study are shown at higher magnification and with the virus names on the right. The viruses isolated in this study are shown in red. All other viruses are color-coded based on the country of isolation (i.e., China, Vietnam, or other). Nodes with bootstrap values greater than 70% are indicated by open diamonds. Bootstrapping converged after 550 replicates. A/turkey/Minnesota/957/80 (H6N6) HA was used to root the phylogenetic tree but is not shown here due to the long branch length.

To generate a phylogenetic tree for the HA genes, all H6 HA sequences collected in Asia between 1 January 1995 and 24 July 2023 were downloaded from GISAID (www.gisaid.org, accessed on 9 September 2023). The HA sequences of these viruses form separate subgroups including the W312 group and Groups I, II, and III [9] (Figure 1). The HA sequences of the H6 viruses characterized here formed several sub-groups within Group II (Figure 1). In one subgroup (shaded in purple in Figure 1), the HA sequences of the Vietnamese H6 viruses we isolated were most closely related to the HA sequences of H6 virus isolates from China. In the other subgroups (shaded in yellow, green, and red in Figure 1), our surveillance sample sequences were similar to the HA sequences of other Vietnamese H6 viruses.

**Figure 2:**
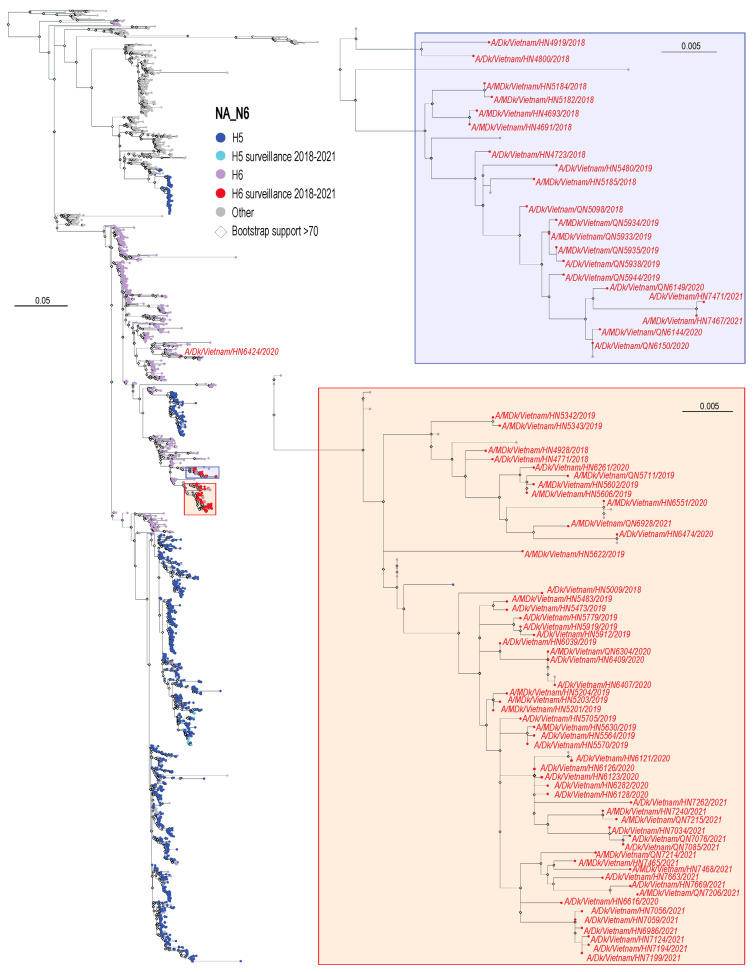
Phylogenetic analysis of the N6 NA genes of H6 influenza viruses isolated in Vietnamese live bird markets from 2018 to 2021. Same as Figure 1, but for N6 NA. Highly pathogenic avian H5 viruses isolated in this study and in our earlier study [31] are shown in light blue. Other H6 and H5 viruses are shown in purple or dark blue, respectively. H9 viruses are shown in orange. All other viruses are shown in gray. Bootstrapping converged after 550 replicates. A/turkey/Minnesota/957/80 (H6N6) NA was used to root the phylogenetic tree but is not shown here due to the long branch length.

For the phylogenetic analysis of the NA genes, we downloaded all N6 NA sequences collected from Asia between 1 January 1995 and 24 July 2023; since only two of the viruses characterized here possessed an NA of the N2 subtype, we did not generate a phylogenetic tree for N2 NAs. The N6 NA sequences of the viruses isolated in our study were similar to each other and most closely related to the NA sequences of other Vietnamese H6N6 viruses, except for the NA sequence of A/duck/Vietnam/HN6424/2020, which grouped with the NA sequences of Chinese H6N6 viruses (shown in red in the left panel of Figure 2). In the phylogenetic tree, the NA sequences of all H5N6 viruses isolated during our surveillance activities in Vietnam are shown (Figure 2, light blue; see [31]). The NA sequences of these viruses are not close to the NA sequences of the H6N6 viruses in the tree, indicating that the NA viral RNA segment was not recently exchanged between these viruses through reassortment.

For the remaining viral RNA segments, all avian influenza virus sequences from Asia collected between 1 January 1995 and 24 July 2023 were used for the phylogenetic analysis. The PB2 sequences (encoding a subunit of the viral polymerase complex) of the H6 viruses isolated in Vietnam from 2018 to 2021 fall into two large subgroups (Figure S1). The PB2 sequences in one subgroup (shaded in blue in Figure S1) are most closely related to the PB2 sequences of previously reported H6 viruses. The PB2 sequences in the other subgroup (shaded in red in Figure S1) are most closely related to the PB2 sequences of highly pathogenic H5 avian influenza viruses (including viruses isolated during our surveillance studies [31]), indicating reassortment among avian influenza viruses.

Most of the H6 PB1 sequences (encoding the PB1 subunit of the viral polymerase complex and the PB1-F2 protein, a minor virulence factor) identified in our study grouped with previously reported H6 PB1 sequences (Figure S2). However, three H6 PB1 sequences (shaded in blue in Figure S2) are more closely related to the PB1 sequences of highly pathogenic H5 viruses, again indicating reassortment among avian influenza viruses.

The PA sequences (encoding the third subunit of the viral polymerase complex and the PA-X protein, another minor virulence factor) of the H6 viruses isolated in our study formed three groups (Figure S3). The first group (shaded in red in Figure S3) is interspersed with the PA sequences of the highly pathogenic H5 viruses described in our earlier publication [31]. In the subgroups shaded in blue and yellow in Figure S3, the PA sequences from our study grouped with the PA sequences of highly pathogenic H5 or other avian influenza viruses, providing additional confirmation of reassortment.

Most of the H6 NP (encoding the viral nucleoprotein) and M (encoding the M1 matrix and M2 ion channel proteins) sequences in our study were very similar to each other and closely related to previously published H6 NP and M sequences, respectively (Figures S4 and S5, respectively), except for three H6 NP sequences that were more closely related to H5, H9, and ‘other’ NP sequences, and three H6 M sequences that were more closely related to ‘other’ M sequences, demonstrating again reassortment between H6 and other avian influenza viruses.

For the NS viral RNA segment (encoding the NS1 non-structural protein and the NEP nuclear export factor), two phylogenetically distinguishable alleles exist. Allele A is found among avian and mammalian influenza A viruses, whereas allele B has been detected exclusively in avian influenza A viruses. Most of the H6 NS sequences analyzed here belonged to allele A (Figure S6). They were very similar to each other and related to previously published H6 NS sequences. Interestingly, we also detected one H6 NS sequence of allele B.

### 3.3. Analysis of H6 Virus Sequences

To analyze the H6 virus sequences in more detail, their consensus sequences were compared with each other (Supplementary Table S4) and with publicly available information. The consensus sequences also served as reference sequences for the identification of viral subpopulations. We limited our analysis to the non-synonymous mutations detected in ≥3% of the sequence reads (Supplementary Table S5).

The viral receptor-binding and fusion protein HA is synthesized as a precursor protein that is post-translationally cleaved into HA1 and HA2 to expose the N-terminus of HA2 (i.e., the fusion peptide), which mediates the fusion between the viral and cellular membranes. Low pathogenic influenza viruses typically encode a single basic amino acid at the HA cleavage site, whereas highly pathogenic influenza viruses encode multiple basic amino acids at this site. The H6 viruses analyzed here have a cleavage site with the sequence QIETR/GLF or QIKTR/GLF (amino acid positions 327–334; the backslash indicates the site of HA cleavage) (Supplementary Table S4), confirming that they are low pathogenic influenza viruses.

The amino acids at several positions of HA (including 137, 190, 222, 225, 226, and 228) have been implicated in the receptor-binding specificity of H6 HAs [24,25,27,28]. At HA amino acid position 226, all viruses isolated here encoded glutamine (Supplementary Table S4), which confers preferential binding to ‘avian-type’ receptors and is typically encoded by avian influenza viruses at this site. Likewise, the Vietnamese H6 viruses characterized here also possessed the ‘avian-type’ amino acid (i.e., glycine) at HA amino acid position 228 (Supplementary Table S4). However, Ni et al. [27] found that H6 HAs encoding HA-228G together with HA-137S and HA-190E bind to both ‘avian-type’ and ‘human-type’ receptors. All H6 viruses characterized here possessed the HA-137S/190E/228G motif (Supplementary Table S4) and therefore may bind to both α2,3- and α2,6-linked sialic acids. A complete switch to ‘human-type’ receptor-binding specificity is conferred by the HA-G225D substitution [28]; however, the viruses studied here all encode HA-225G.

The NA gene encodes the neuraminidase protein which cleaves the glycosidic linkages of neuraminic acids to facilitate influenza virus release from infected cells. The catalytic residues (R118, D151, R152, R224, E276, R292, R371, Y406) [38] were conserved in the viruses analyzed here. The NA proteins of some avian influenza viruses also have a second catalytic site, formed by S367, S370, S372, and W403 [39]. These residues were also conserved in the viruses analyzed here (Supplementary Table S4). The neuraminidase protein is the target of approved anti-influenza compounds. The viruses characterized in our study do not encode NA-R292K or -E119V+I222L (Supplementary Table S4), which confer resistance to neuraminidase inhibitors.

The viral polymerase basic 2 (PB2) protein is part of the viral polymerase complex and a major determinant of influenza virus host range restriction and pathogenicity [40], but major markers of mammalian-adaptation [i.e., the PB2-E627K [41,42] and -D701N [43] substitutions] and other substitutions that affect virulence [i.e., PB2-I147T/K339T/A588T [44], -E158G [45], -T271A [46], -Q590S [47], -Q591K/R [47]] were not detected in the H6 viruses we analyzed (Supplementary Table S4). The PB2-S714R substitution is known to increase the viral polymerase activity and virulence of H7N7 and H5N1 viruses [48,49,50,51]; several of the H6 viruses analyzed here encode glycine at position 714 of PB2 (Supplementary Table S4), but the consequences of this substitution are not known. Several studies have indicated that the amino acid at PB2-292 affects replication, disease severity, and transmission [52,53,54]. Some of the H6 viruses isolated in Vietnam encode PB2-292V, which confers higher polymerase activity in human cells and is important for the transmissibility of the H7N9 virus in guinea pigs.

The viral polymerase basic 1 (PB1) protein encodes the viral RNA-dependent RNA polymerase and is another component of the influenza virus polymerase complex. Several substitutions in this protein can affect the viral polymerase activity, including PB1-E180D [55], -T296R [56], -V473L [57], -K480R [58], -S524G [59], -K577E [60], and -P598L [57]; however, none of these substitutions were detected in the H6 viruses in our study (Supplementary Table S4). In contrast, several of the H6 viruses isolated in our study encoded PB1-317V, which, in one study, increased the polymerase activity of an H5 influenza virus in chicken cells compared to PB1-317M [55].

The third component of the influenza virus polymerase complex, the polymerase acidic protein PA, is the target of the antiviral compound baloxavir. The H6 virus PA proteins analyzed here do not encode PA-I38T/M/F substitutions (Supplementary Table S4), which confer resistance to baloxavir [61]. Computational analyses have identified several amino acids in PA that are host-specific [62,63,64,65,66]. Most of the H6 viruses isolated in our study encoded amino acids typically found in avian influenza viruses, but five isolates contained human virus-specific residues, namely, PA-55N (A/duck/Vietnam/HN4780/2018, A/duck/Vietnam/HN4776/2018), PA-57Q (A/duck/Vietnam/HN5480/2019), PA-268I (A/Muscovy duck/Vietnam/HN5622/2019), and PA-404S and -409N (A/duck/Vietnam/HN6424/2020).

The viral nucleoprotein, NP, also possesses amino acids that are characteristic of avian or human influenza viruses, respectively, based on computational analyses [62,63,64,65,66,67]. Some of the H6 viruses encoded a human virus-characteristic amino acid, specifically, NP-33I (A/Muscovy duck/Vietnam/HN5182/2018, A/Muscovy duck/Vietnam/HN5185/2018, and A/Muscovy duck/Vietnam/HN5187/2018), NP-127D (several H6 viruses), and NP-214K (most H6 viruses) (Supplementary Table S4).

The M viral RNA segment encodes the matrix, M1, protein, and major structural component of the virion. The M1-I15V substitution detected in A/duck/Vietnam/QN6076/2020, A/duck/Vietnam/HN6261/2020, A/duck/Vietnam/HN6270/2020, and A/duck/Vietnam/QN6085/2020 and the M1-F144L substitution encoded by A/duck/Vietnam/HN4919/2018 (Supplementary Table S4) conferred high growth properties in a previous study to the live attenuated A/Leningrad/134/17/1957 strain [68], indicating that M1-144L plays an important role in the viral life cycle. Very few sequence nucleotide polymorphisms were detected in the M1 coding region, but the M1-A209T substitution detected in 19.6% of the sequence reads of A/duck/Vietnam/HN5705/2019 (Supplementary Table S5) may have a biological functional, given that the M1-T209A substitution was found to affect the morphology and reduce the virus titers of a human pandemic H1N1 virus in human cells [69]. The M viral RNA segment also encodes the ion channel M2 protein, the target of the antiviral compounds amantadine and rimantadine. Amino acid substitutions at M2 amino acid positions 26, 27, 30, or 31 can confer resistance to these antiviral compounds, but none of the substitutions known to render M2 resistant to the ion channel blockers is encoded by the virus assessed here (Supplementary Table S4).

As stated earlier, all but one of the H6 viruses analyzed here encoded an allele A NS viral RNA segment (Supplementary Table S4). The amino acid at position 48 of NS1 affects the levels of cellular mRNA translation [70], with most viruses in our study encoding NS1-48S, which confers a more efficient translation of cellular mRNAs compared to NS1-48N in in vitro translation assays.

The PB1-F2 protein (encoded by an alternate open reading frame in the PB1 viral RNA segment) also affects host interferon responses; in addition, this protein modulates the severity of bacterial infections, the induction of apoptosis, and inflammasome function (reviewed in [67,68]). Except for three viruses, all H6 analyzed in our study encoded truncated PB1-F2 proteins of 34 amino acids in length, resulting from a mutation that creates a premature stop codon (Supplementary Table S4). None of the full-length PB1-F2 proteins encoded PB1-F2-N66S, which increases influenza virulence [71].

## 4. Discussion

Reassortment plays an important role in the ecology of influenza viruses. Co-infections of birds with different influenza viruses can occur where large numbers of animals are in close proximity, such as congregation sites, poultry production farms, and live bird markets. One study found that in more than 80% of the poultry flocks tested, more than one influenza virus strain was detected [72], indicating ample opportunity for reassortment. Another study in poultry farms identified co-infections with two different influenza virus strains in 2.6–9.1% of the flocks [73]. Interestingly, a study that modelled the co-infection potential of poultry flocks with H5N1 and H9N2 viruses found that the distance to live bird markets was the strongest predictor of co-infection [74], suggesting that live bird markets may also play a role in co-infections in poultry flocks. Between 2000 and 2006, Pepin et al. [75] collected more than 42,000 samples from live bird markets and farms in southern China and, based on HI titers, found a co-infection rate of approximately 11%. Most of these co-infections occurred in ducks [75]. Similarly, in live bird markets in Cambodia, 0.8% of ducks and 4.5% of chickens were found to be co-infected, primarily with H5 and H9 viruses [76]. Based on our analysis of whole genome deep-sequencing data, we here found that more than half of the H6 samples contained sequences from more than one influenza virus strain. In addition to the high rate of samples containing sequences from more than one influenza virus strain, our phylogenetic analyses also indicated reassortment between H6 and other avian influenza viruses. The close proximity of animals coupled with the constant introduction of naïve and infected birds into live bird markets provides ideal conditions for infections with different influenza viruses and subsequent reassortment events.

The H6 viruses characterized here encoded a single basic amino acid at the HA cleavage site, consistent with low pathogenicity in chickens. However, they also encoded an amino acid motif in HA (HA-137S/190E/228G) that confers binding to both α2,3- and α2,6-linked sialic acids [27]. The dual receptor-binding specificity of H6 viruses may, in part, explain their ability to infect mammals. Although only one human infection with an H6 virus has been documented [4], many more cases may have occurred. An analysis of sera from veterinarians and healthy controls in the US showed that some veterinarians exposed to birds have low antibody titers to H5, H6, and/or H7 influenza viruses [77]. Another study of more than 15,600 sera collected in 2009–2011 in China tested for antibodies to a representative H6 virus. The overall seropositivity rate was low (<1%); however, workers in live poultry markets were more likely to have antibodies to the H6 virus than workers in large-scale poultry farms, and exposure to live bird markets was a risk factor for serum-positivity to H6 [78]. These findings indicate that human infections with H6 viruses may occur more frequently than reported. Together, our data indicate that the H6 viruses circulating in Vietnam should be monitored for their evolution and propensity to infect humans.

## Data Availability

The original contributions presented in the study are included in the article/supplementary material, and further inquiries can be directed to the corresponding authors.

## Supplementary Materials

The following supporting information can be downloaded at: https://www.mdpi.com/article/10.3390/v16030367/s1, Figure S1. Phylogenetic analysis of the PB2 genes of H6 influenza viruses isolated in Vietnamese live bird markets from 2018 to 2021; Figure S2. Phylogenetic analysis of the PB1 genes of H6 influenza viruses isolated in Vietnamese live bird markets from 2018 to 2021; Figure S3. Phylogenetic analysis of the PA genes of H6 influenza viruses isolated in Vietnamese live bird markets from 2018 to 2021; Figure S4. Phylogenetic analysis of the NP genes of H6 influenza viruses isolated in Vietnamese live bird markets from 2018 to 2021; Figure S5. Phylogenetic analysis of the M genes of H6 influenza viruses isolated in Vietnamese live bird markets from 2018 to 2021; Figure S6. Phylogenetic analysis of the NS genes of H6 influenza viruses isolated in Vietnamese live bird markets from 2018 to 2021; Table S1: Accession numbers; Table S2: Acknowledgement of sequences obtained from GISAID; Table S3: Identical sequences not shown in trees; Table S4: Consensus sequences; Table S5: Subpopulations.
