# Supplementary figures and images for: Avian H6 Influenza Viruses in Vietnamese Live Bird Markets during 2018–2021
Source: Viruses. 2024 Feb 27;16(3):367. doi: 10.3390/v16030367 (PMC10975462; doi:10.3390/v16030367)

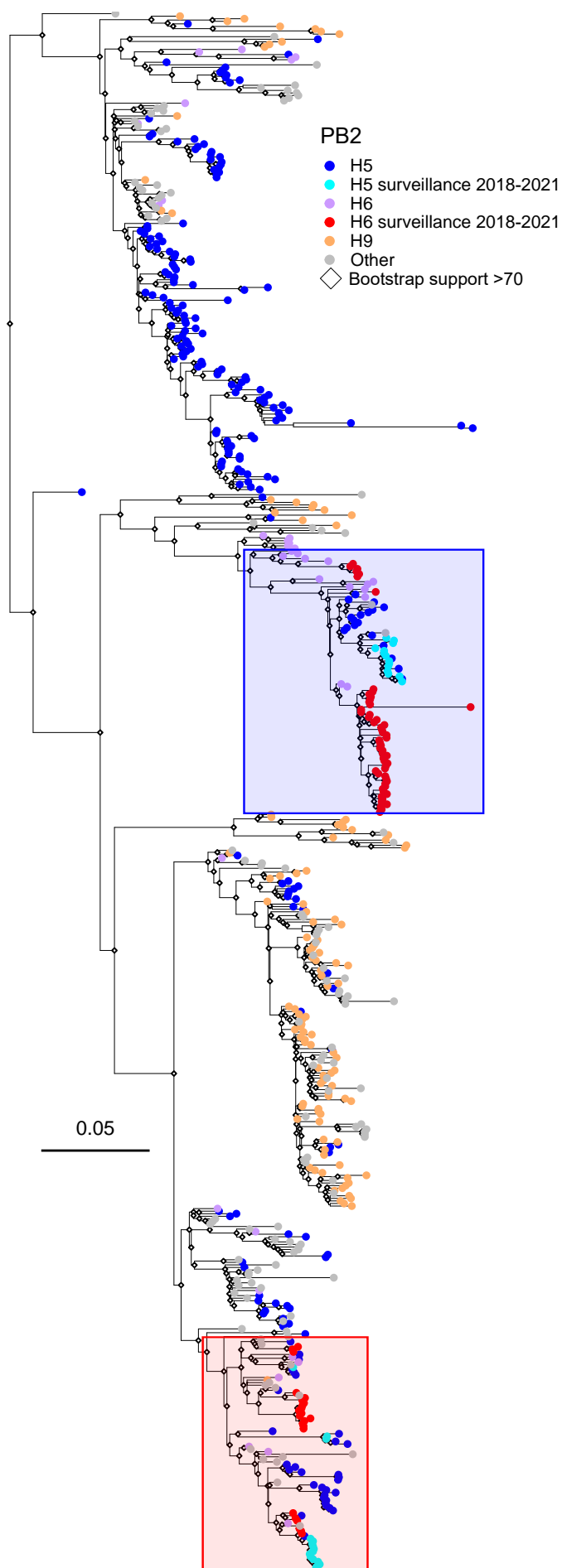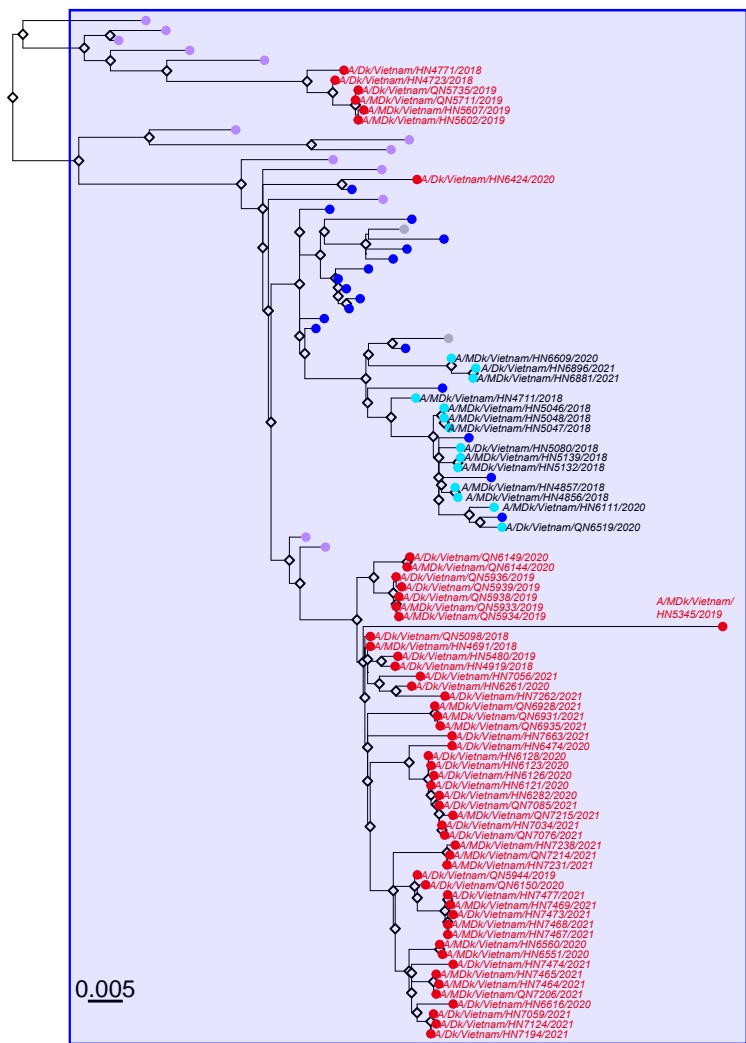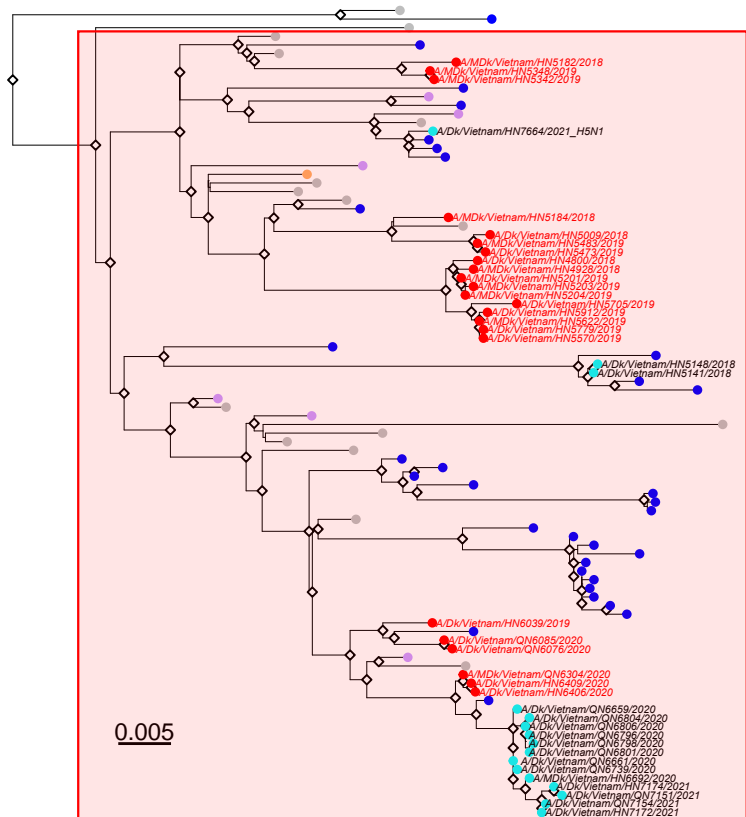

Supplement: Supplementary file 1 [file viruses-16-00367-s001.zip › Supplementary files/Figure S1 - PB2 tree - 01-05-24.pdf]

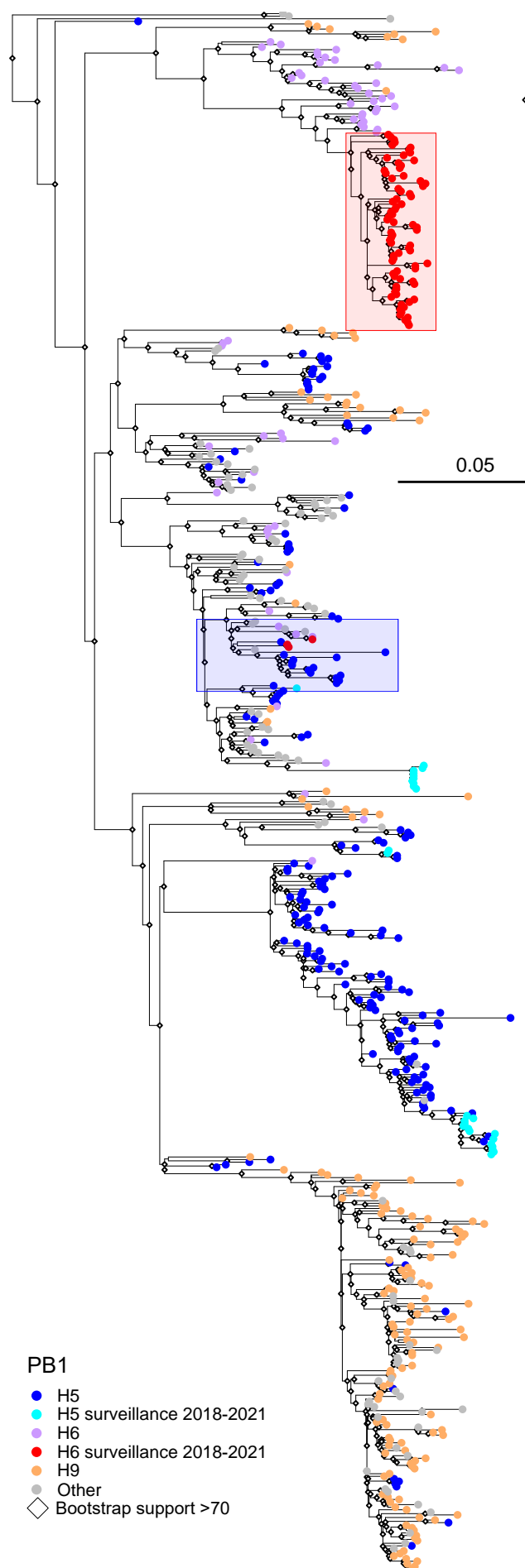

PB1

- H5
- H5 surveillance 2018-2021
- H6
- H6 surveillance 2018-2021
- H9
- Other
- ◇ Bootstrap support >70

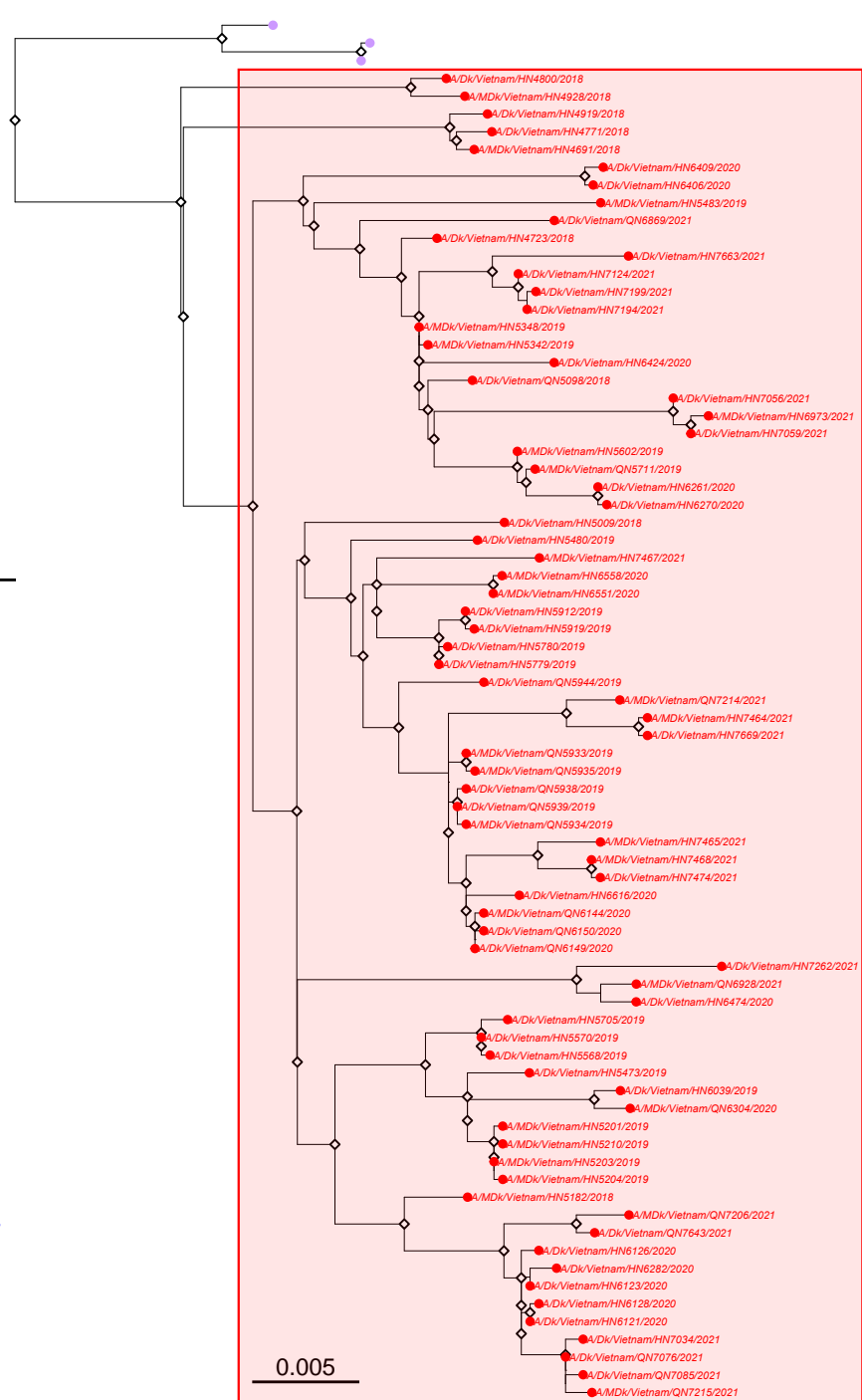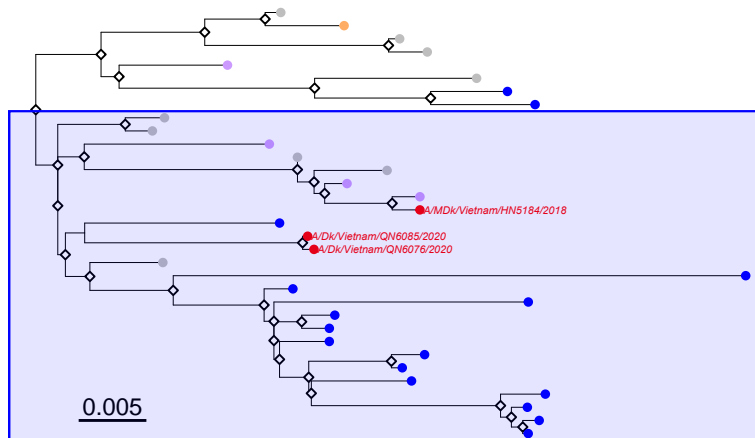

Supplement: Supplementary file 1 [file viruses-16-00367-s001.zip › Supplementary files/Figure S2 - PB1 tree - 01-05-24.pdf]

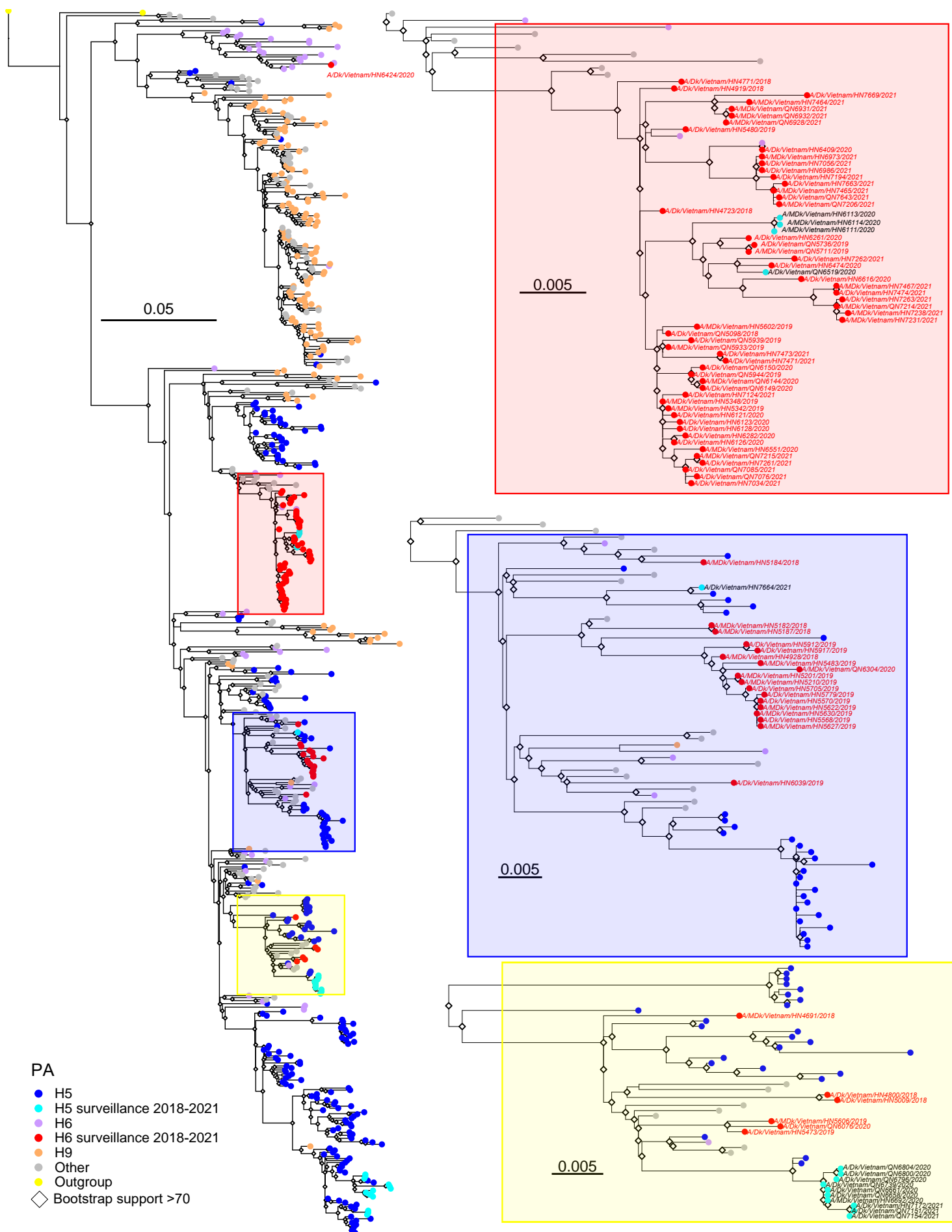

Supplement: Supplementary file 1 [file viruses-16-00367-s001.zip › Supplementary files/Figure S3 - PA tree - 01-05-24.pdf]

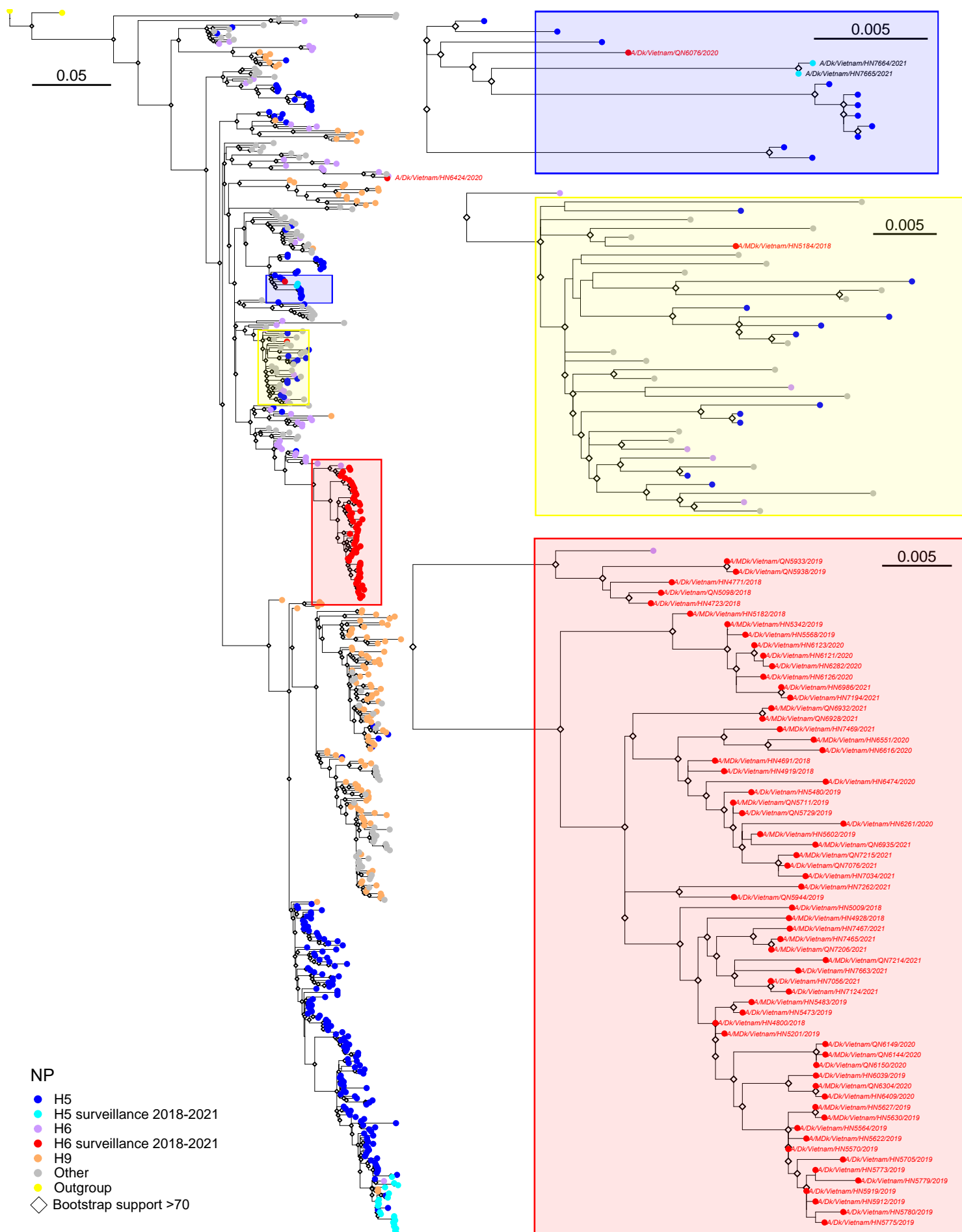

Supplement: Supplementary file 1 [file viruses-16-00367-s001.zip › Supplementary files/Figure S4 - NP tree - 01-05-24.pdf]

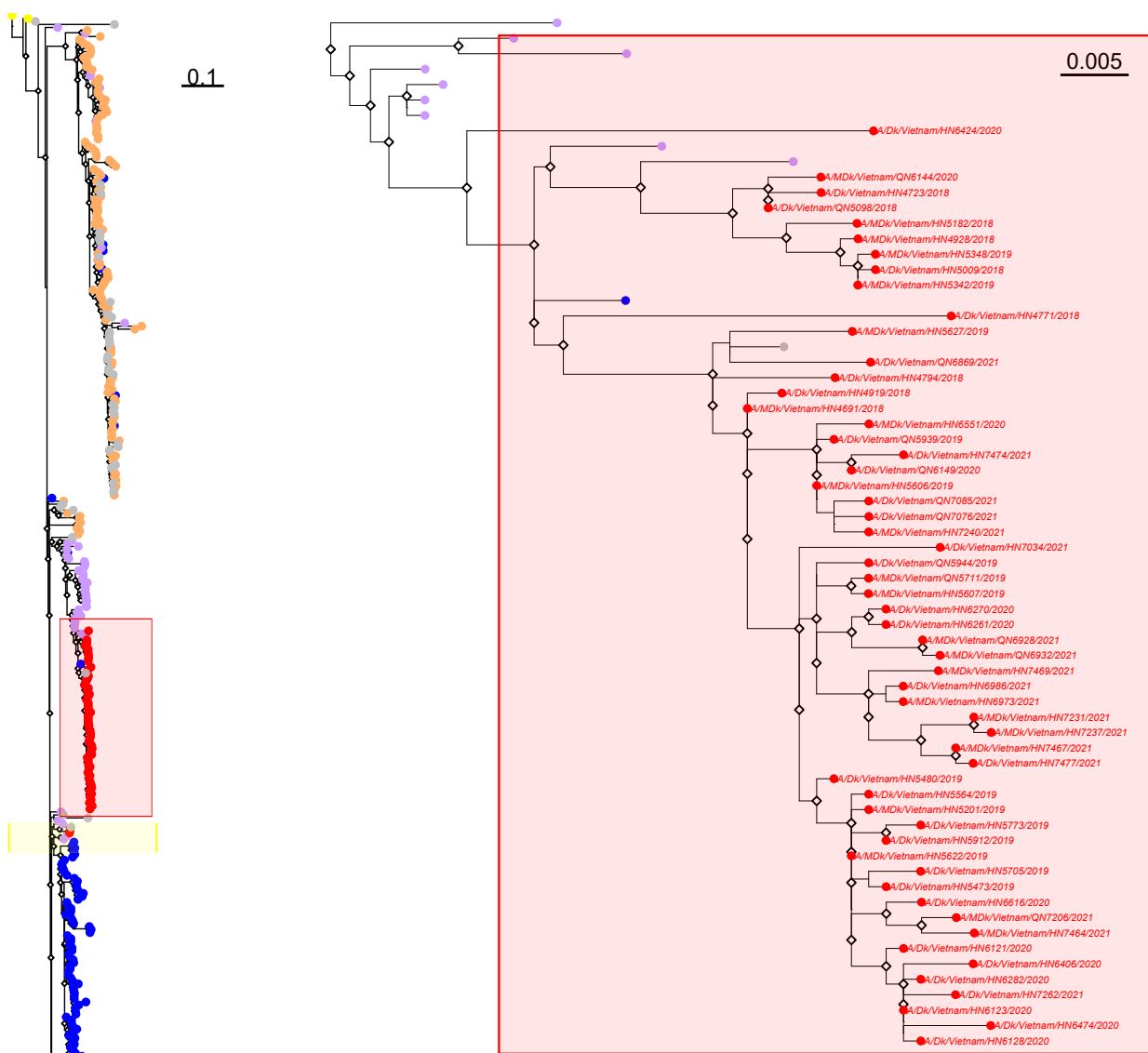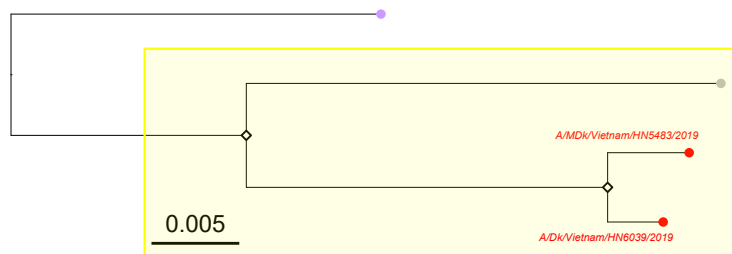

NS

- H5
- H5 surveillance 2018-2021
- H6
- H6 surveillance 2018-2021
- H9
- Other
- Outgroup
- ◇ Bootstrap support >70

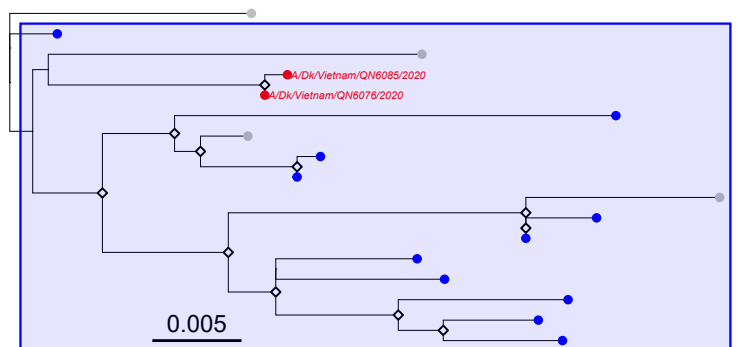

A/MDk/Vietnam/  
 HN5184/2018

Supplement: Supplementary file 1 [file viruses-16-00367-s001.zip › Supplementary files/Figure S6 - NS tree - 01-05-24.pdf]
