# Supplementary material for: Avian H6 Influenza Viruses in Vietnamese Live Bird Markets during 2018–2021
Source: Viruses. 2024 Feb 27;16(3):367. doi: 10.3390/v16030367 (PMC10975462; doi:10.3390/v16030367)
Supplement: Supplementary file 1 [file Figure S5 - M tree - 01-05-24.pdf › Supplementary files/Figure S5 - M tree - 01-05-24.pdf]

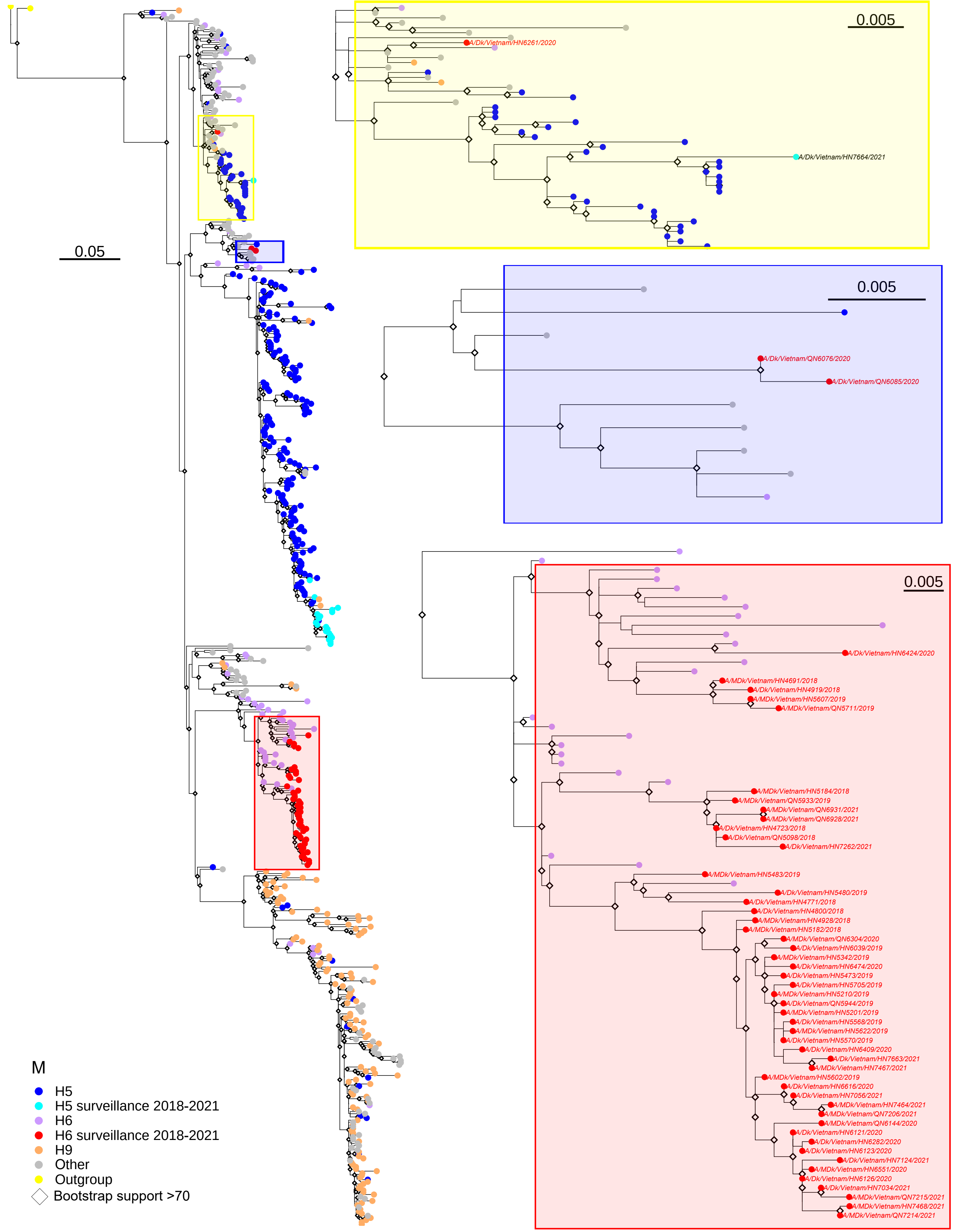

0.05
0.005
A/Dk/Vietnam/HN6261/2020
A/Dk/Vietnam/HN7664/2021
0.005
A/Dk/Vietnam/QN6076/2020
A/Dk/Vietnam/QN6085/2020
0.005
A/Dk/Vietnam/HN6424/2020
A/MDk/Vietnam/HN4691/2018
A/Dk/Vietnam/HN4919/2018
A/MDk/Vietnam/HN5607/2019
A/MDk/Vietnam/QN5711/2019
A/MDk/Vietnam/HN5184/2018
A/MDk/Vietnam/QN5933/2019
A/MDk/Vietnam/QN6931/2021
A/MDk/Vietnam/QN6928/2021
A/Dk/Vietnam/HN4723/2018
A/Dk/Vietnam/QN5098/2018
A/Dk/Vietnam/HN7262/2021
A/MDk/Vietnam/HN5483/2019
A/Dk/Vietnam/HN5480/2019
A/Dk/Vietnam/HN4771/2018
A/Dk/Vietnam/HN4800/2018
A/MDk/Vietnam/HN4928/2018
A/MDk/Vietnam/HN5182/2018
A/MDk/Vietnam/QN6304/2020
A/Dk/Vietnam/HN6039/2019
A/MDk/Vietnam/HN5342/2019
A/Dk/Vietnam/HN6474/2020
A/Dk/Vietnam/HN5473/2019
A/Dk/Vietnam/HN5705/2019
A/MDk/Vietnam/HN5210/2019
A/Dk/Vietnam/QN5944/2019
A/MDk/Vietnam/HN5201/2019
A/Dk/Vietnam/HN5568/2019
A/MDk/Vietnam/HN5622/2019
A/Dk/Vietnam/HN5570/2019
A/Dk/Vietnam/HN6409/2020
A/Dk/Vietnam/HN7663/2021
A/MDk/Vietnam/HN7467/2021
A/MDk/Vietnam/HN5602/2019
A/Dk/Vietnam/HN6616/2020
A/Dk/Vietnam/HN7056/2021
A/MDk/Vietnam/HN7464/2021
A/MDk/Vietnam/QN7206/2021
A/MDk/Vietnam/QN6144/2020
A/Dk/Vietnam/HN6121/2020
A/Dk/Vietnam/HN6282/2020
A/Dk/Vietnam/HN6123/2020
A/Dk/Vietnam/HN7124/2021
A/MDk/Vietnam/HN6551/2020
A/Dk/Vietnam/HN6126/2020
A/Dk/Vietnam/HN7034/2021
A/MDk/Vietnam/QN7215/2021
A/MDk/Vietnam/HN7468/2021
A/MDk/Vietnam/QN7214/2021
M
H5
H5 surveillance 2018-2021
H6
H6 surveillance 2018-2021
H9
Other
Outgroup
Bootstrap support >70
